# Redox mediators for high-performance lithium–oxygen batteries

**DOI:** 10.1093/nsr/nwac040

**Published:** 2022-03-04

**Authors:** Yaying Dou, Zhaojun Xie, Yingjin Wei, Zhangquan Peng, Zhen Zhou

**Affiliations:** Engineering Research Center of Advanced Functional Material Manufacturing of Ministry of Education, School of Chemical Engineering, Zhengzhou University, Zhengzhou 450001, China; Institute of New Energy Material Chemistry, School of Materials Science and Engineering, Nankai University, Tianjin 300350, China; Key Laboratory of Physics and Technology for Advanced Batteries (Ministry of Education), College of Physics, Jilin University, Changchun 130012, China; Laboratory of Advanced Spectro-electrochemistry and Li-ion Batteries, Dalian Institute of Chemical Physics, Chinese Academy of Sciences, Dalian 116023, China; Engineering Research Center of Advanced Functional Material Manufacturing of Ministry of Education, School of Chemical Engineering, Zhengzhou University, Zhengzhou 450001, China

**Keywords:** Li–O2 batteries, redox mediators, catalysts, oxygen reduction reaction, oxygen evolution reaction

## Abstract

Aprotic lithium–oxygen (Li–O_2_) batteries are receiving intense research interest by virtue of their ultra-high theoretical specific energy. However, current Li–O_2_ batteries are suffering from severe barriers, such as sluggish reaction kinetics and undesired parasitic reactions. Recently, molecular catalysts, i.e. redox mediators (RMs), have been explored to catalyse the oxygen electrochemistry in Li–O_2_ batteries and are regarded as an advanced solution. To fully unlock the capability of Li–O_2_ batteries, an in-depth understanding of the catalytic mechanisms of RMs is necessary. In this review, we summarize the working principles of RMs and their selection criteria, highlight the recent significant progress of RMs and discuss the critical scientific and technical challenges on the design of efficient RMs for next-generation Li–O_2_ batteries.

## Teaser

This review provides the operation and design principles, latest development, and future challenges and perspectives of redox mediators, which demonstrate a promising strategy to solve the issues in Li–O_2_ batteries.

## INTRODUCTION

Human beings are being confronted with significant challenges, such as the excessive depletion of non-renewable fossil fuels and increasingly serious climate change. To secure safe and sustainable energy supply, various green and renewable energies (such as solar, wind and tidal energy) have been exploited. However, these energy sources are intermittent and the peak time of electricity generation and demand is often mismatched. Therefore, tremendous efforts have been devoted to exploring novel energy conversion and storage systems (Li–S [1], Li–O_2_ [2,3], Zn–air [4], etc.), with the hope of realizing higher energy density and longer lifetime than state-of-the-art Li-ion batteries. Among these technologies beyond Li-ion batteries, aprotic lithium–oxygen (Li–O_2_) batteries have attracted much attention because of their unbeatable theoretical specific energy (3500 Wh kg^–1^). This high specific energy results from the electrochemical reaction betwee n oxygen and lithium, $2{\rm{L}}{{\rm{i}}^ + } + {{\rm{O}}_2} + {\rm{\ }}2{{\rm{e}}^ - } = {\rm{L}}{{\rm{i}}_2}\ \!{{\rm{O}}_2}$, which does not involve any heavy transition metals. Furthermore, the use of an environmentally friendly and unlimited source of oxygen makes this battery more attractive as a potentially transformative energy-storage technology.

However, current Li–O_2_ batteries are suffering from many significant challenges, including but not limited to low-rate capability, poor round-trip efficiency and miserable cycle life. These issues are mainly related to the oxygen reactions occurring in the air cathode of Li–O_2_ batteries. For instance, the discharged product Li_2_O_2_, an insulator with a large band gap (∼4.9 eV), often impedes electron transfer and ion diffusion, leading to sluggish kinetics of oxygen electrochemistry. Besides, oxygen species (O_2_^–^, LiO_2_ and ^1^O_2_) formed from oxygen electrochemistry are highly reactive and can react with electrolytes and cathode components producing parasitic side-reaction products (such as Li_2_CO_3_ and LiOH) that further deteriorate the battery performance. To speed up the oxygen electrochemistry in Li–O_2_ batteries, extensive solid catalysts have been proposed, including carbon-based materials, transition metal compounds and noble metals [5–7]. However, traditional solid catalysts frequently encounter certain intractable challenges, such as the high cost of raw materials and complex synthesis, poor solid–solid contact between catalysts and reactants, and aggravated degradation of electrolytes. To achieve the reversible formation and decomposition of Li_2_O_2_ and eliminate the undesired parasitic side reactions, new concepts of catalysis and catalyst design are urgently needed. Recently, soluble redox mediators (RMs), molecular siblings of solid catalysts, have demonstrated outstanding performance in ameliorating sluggish kinetics and enhancing energy efficiency [8,9]. Specifically, RMs act as electron-hole ‘carriers’ to facilitate the electrochemical reactions of Li–O_2_ batteries by transferring electrons between O_2_/Li_2_O_2_ and cathodes. This novel catalysis not only enlarges the reaction region but also suppresses parasitic reactions. Relevant research is in full swing towards building practical RMs-assisted Li–O_2_ batteries.

A few insightful reviews on RMs have been published from various perspectives, offering new opportunities for researchers to explore Li–O_2_ batteries [10–14]. Recently, there has been prominent progress in understanding the catalytic mechanism and the robustness of RMs, the synergy of RMs with other battery components and the reaction kinetics of Li_2_O_2_ with RMs. A comprehensive picture of RMs-assisted Li–O_2_ electrochemistry and a timely update on the progress in this field are essential in the ongoing development of Li–O_2_ batteries. In this review, we systematically and comprehensively summarize the recently updated development and application of RMs in Li–O_2_ batteries. Specifically, we first introduce the fundamental operation and design principles of RMs and the latest development associated with RMs; then highlight the challenges encountered in the application of RMs in Li–O_2_ batteries; and finally conclude with perspectives on the remaining knottiness and future research opportunities towards making effective RMs in Li–O_2_ batteries.

## FUNDAMENTAL PRINCIPLES

### Working mechanisms

**Figure 1.**
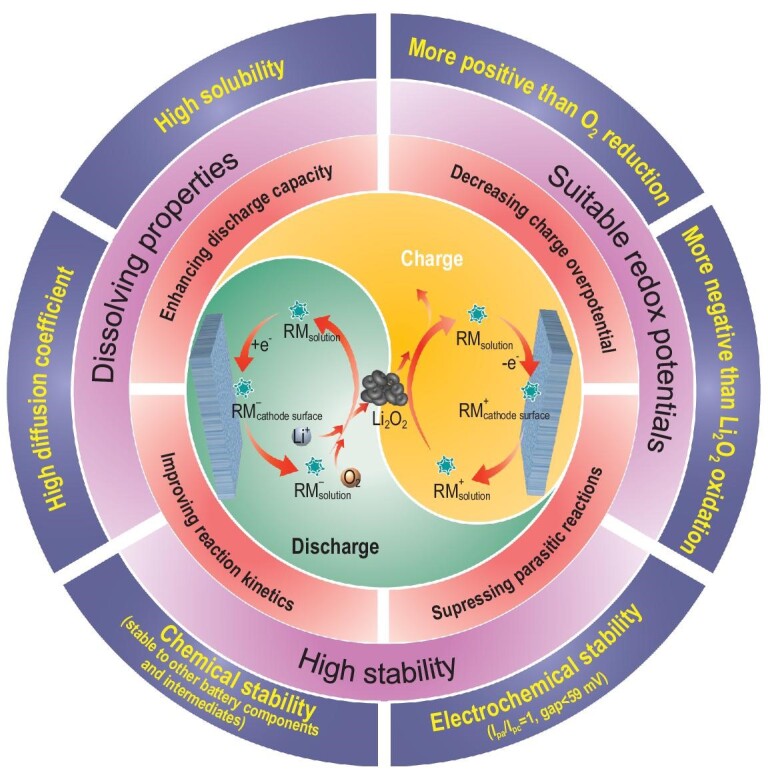
Working mechanisms, advantages and critical criteria of RMs in Li–O_2_ batteries.

RMs are electrochemically active species that facilitate the oxygen reduction reaction (ORR) and oxygen evolution reaction (OER) in Li–O_2_ batteries. They participate in the operation of Li–O_2_ batteries through the following mechanisms, as illustrated in Fig. 1.

#### ORR process

In Equation (1), RM diffuses from the solution to the cathode surface:

(1) \begin{equation*} {\rm{R}}{{\rm{M}}_{{\rm{solution}}}} \to {\rm{R}}{{\rm{M}}_{{\rm{cathode\ surface}}}}.\end{equation*}

In Equation (2), RM is electrochemically reduced to RM^–^ at the cathode surface:

(2) \begin{equation*}{\rm{R}}{{\rm{M}}_{{\rm{cathode\ surface}}}}{\rm{\ + \ }}{{\rm{e}}^{\rm{ - }}} \to {\rm{\ RM}}_{{\rm{cathode\ surface}}}^{\rm{ - }}.\end{equation*}

In Equation (3), RM^–^ diffuses from the cathode surface to the solution:

(3) \begin{equation*}{\rm{RM}}_{{\rm{cathode\ surface}}}^{\rm{ - }}{\rm{\ }} \to {\rm{\ RM}}_{{\rm{solution}}}^{\rm{ - }}.\end{equation*}

In Equation (4), O_2_ is chemically reduced by RM^–^ and then combines with Li^+^ to form Li_2_O_2_ suspended in solutions:

(4) \begin{equation*}2{\rm{RM}}_{{\rm{solution}}}^{\rm{ - }}{\rm{\ + \ }}{{\rm{O}}_{\rm{2}}}{\rm{\ + \ 2L}}{{\rm{i}}^{\rm{ + }}}{\rm{\ }} \to {\rm{\ 2RM\ + \ L}}{{\rm{i}}_{\rm{2}}}{{\rm{O}}_{\rm{2}}}.\end{equation*}

#### OER process

In Equation (5), RM diffuses from the solution to the cathode surface:

(5) \begin{equation*}{\rm{R}}{{\rm{M}}_{{\rm{solution}}}}{\rm{\ }} \to {\rm{\ R}}{{\rm{M}}_{{\rm{cathode\ surface}}}}.\end{equation*}

In Equation (6), RM is electrochemically oxidized to RM^+^ at the cathode surface:

(6) \begin{equation*}{\rm{R}}{{\rm{M}}_{{\rm{cathode\ surface}}}}{\rm{\ - \ }}{{\rm{e}}^{{\rm{ - \ }}}} \to {\rm{\ RM}}_{{\rm{cathode\ surface}}}^{\rm{ + }}.\end{equation*}

In Equation (7), RM^+^ diffuses from the cathode surface to the solution:

(7) \begin{equation*}{\rm{RM}}_{{\rm{cathode\ surface}}}^{\rm{ + }}{\rm{\ }} \to {\rm{\ RM}}_{{\rm{solution}}}^{\rm{ + }}.\end{equation*}

In Equation 8, RM^+^ chemically oxidizes Li_2_O_2_ with O_2_ evolution:

(8) \begin{equation*}{\rm{2RM}}_{{\rm{solution}}}^{\rm{ + }}{\rm{\ + \ L}}{{\rm{i}}_{\rm{2}}}{{\rm{O}}_{{\rm{2\ }}}} \to {\rm{\ 2RM\ + \ }}{{\rm{O}}_{\rm{2}}}{\rm{ + \ 2L}}{{\rm{i}}^{\rm{ + }}}.\end{equation*}

These reactions illustrate that RMs do not change the net ORR or OER reactions, but alter the specific reaction pathways. Upon discharge, RMs are electrochemically reduced prior to O_2_, followed by chemical reduction of O_2_ by RM^–^ to Li_2_O_2_ in electrolytes. This process delays the formation of insulating Li_2_O_2_ films through the surface-mediated ORR, thereby enhancing the discharge capacity. Upon charge, RMs are preferentially oxidized electrochemically at cathode surfaces and then diffuse to electrolytes, where they chemically oxidize Li_2_O_2_ with O_2_ evolution. In this case, regardless of the location, size and structure of Li_2_O_2_, the dissolved RMs can guarantee feasible wet contact with them. To this end, the catalytic functionality of RMs can be exerted on all the Li_2_O_2_ products, with an entire decomposition and a relatively low charge overpotential. Moreover, this electrocatalytic mechanism of electron transfer followed by chemical reaction (EC) can circumvent the formation of highly oxidative oxygen species, such as singlet oxygen (^1^O_2_) and superoxide species (O_2_^–^ and LiO_2_), and can efficaciously suppress the degradation of electrolytes and electrodes of Li–O_2_ batteries [15].

### Critical criteria for selecting RMs

To realize a Li–O_2_ battery with high capacity and long lifespan, RMs must meet the following conditions (Fig. 1): (i) their electrochemical redox potentials should be close to the thermodynamic equilibrium potential (2.96 V versus Li/Li^+^) of Li–O_2_ batteries; (ii) they should have enough high solubility with rapid mass transport (diffusion coefficient, *D_t_*) in electrolytes; (iii) they must have high electrochemical and chemical stability during operation. Materials that fulfill these requirements can serve as potential RMs for Li–O_2_ batteries.

To efficiently reduce O_2_ or oxidize Li_2_O_2_, an ideal RM should have an equilibrium potential more negative than O_2_ reduction or positive than Li_2_O_2_ oxidation from the aspect of thermodynamics. Essentially, the redox potentials of RMs determine the charge and discharge potentials of batteries; therefore, they should be as close to 2.96 V as possible to improve the round-trip efficiency of Li–O_2_ batteries. Generally, redox potentials are intrinsic properties, which depend on the type of active centers, functional groups and chemical substituents. However, the redox potentials of RMs in batteries are also affected by the extrinsic environment, such as solvent type and salt concentration.

As RMs are homogeneously dissolved in electrolytes, high solubility is necessary. Besides, RMs are present in a much lower concentration than Li^+^ in electrolytes; therefore, the mass transport of RMs is mainly driven by diffusion. The diffusion coefficients (*D_t_*) of RM, RM^red^ and RM^ox^ are approximately equal because of their similar chemical structures. Consequently, a preferred RM should also have a high diffusion coefficient to ensure that it can reach more reaction regions in a short time and achieve fast redox-reaction kinetics.

Due to the critical role of RMs in Li–O_2_ batteries, their degradation would be even more detrimental than electrolytes and electrodes. Hence, high electrochemical and chemical stability is indispensable. Typically, ideal RMs should have high electrochemical reversibility with a peak current ratio (i.e. *I*_pa_/*I*_pc_ determined by cyclic voltammetry, CV) as close as 1 and the peak separation should be small (∼59 mV). Meanwhile, a perfect RM and its reduced and oxidized form should have negligible reactivity towards other battery components, such as lithium metal anodes, electrolytes (salts and solvents) and electrode components (active and conducting materials, current collectors and binders). Moreover, RMs should have high chemical stability against attack by highly reactive oxygen intermediates (^1^O_2_, O_2_^–^ and LiO_2_).

## APPLICATION OF RMS TO LI–O_2_ BATTERIES

### RMs for discharge

**Figure 2.**
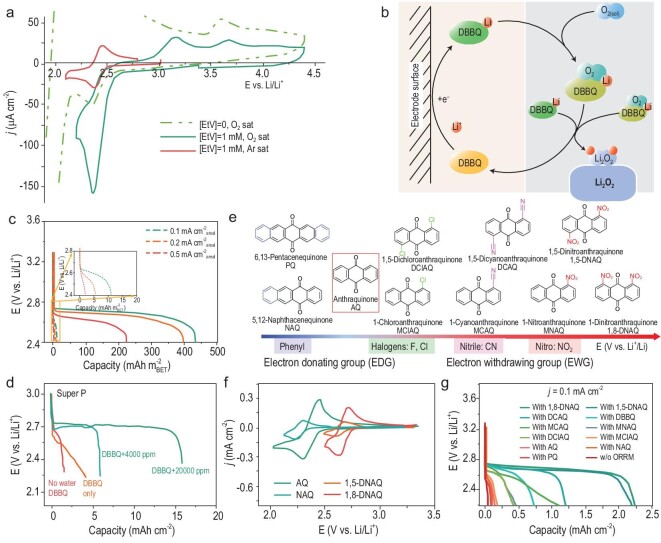
(a) CV curves of BMPTFSI without (dashed line) and with 1 mM EtV(OTf)_2_ under different atmospheres, reproduced from Ref. [18]. (b) Schematic of DBBQ-containing discharge process of the Li–O_2_ battery. Reprinted with permission from Ref. [9]. Copyright 2016 Nature Publishing Group. (c) Discharge curves with (solid lines) and without (dashed lines) DBBQ in TEGDME electrolytes based on the data reproduced from Ref. [9]. (d) Galvanostatic discharge curves of batteries with super P electrodes either with 0.25 M LiTFSI/DME electrolyte, with only DBBQ added, or with both DBBQ and H_2_O added to the neat electrolyte. The data are reproduced from Ref. [19]. (e) Structures of AQ and its derivatives; CV curves of different ORR RMs under Ar atmosphere (f) and discharge curves of the Li–O_2_ batteries with different RMs (g). The data are reproduced from Ref. [23]. BMPTFSI, 1-butyl-3-methylpyrrolidinium bis(trifluoromethylsulfonyl)imide; TEGDME, tetraethylene glycol dimethyl ether.

Currently, the energy density of Li–O_2_ batteries is far below their theoretical promise, which is mainly caused by the deposition of film-like Li_2_O_2_ on electrode surfaces. Although the electrolytes or salts with high donor numbers can promote the growth of Li_2_O_2_ in solutions, these systems are usually vulnerable to reduced oxygen species, particularly LiO_2_, which is inevitable in the traditional ORR pathway. Besides, the phase-transfer catalyst, typically water, can also increase the discharge capacity. Specifically, water would alter the reaction pathway to a single two-electron-transfer process (O_2_ → O_2_^2–^) with a soluble hydroperoxide (HO_2_^–^) intermediate. As a result, the solution-route ORR is significantly triggered and the morphology of Li_2_O_2_ products changes from a toroidal shape to a lamellar one [16,17]. However, excessive moisture may lead to parasitic reactions with Li anodes and cause safety issues. Alternatively, RMs have the capability to address these problems. Lacey and co-workers first proposed an ethyl viologen ditriflate EtV(OTf)_2_ RM to improve the discharge performance of Li–O_2_ batteries [18]. During discharge, EtV(OTf)_2_ acted as a redox shuttle to transfer electrons from the electrode to O_2_, which then formed O_2_^−^, followed by disproportionation to Li_2_O_2_. Regretfully, due to the relatively low redox potential of EtV(OTf)_2_ (2.4 V vs. Li/Li^+^), the parallel direct O_2_ reduction could not be eliminated and the intrinsic problem of electrode passivation remained (Fig. 2a). In contrast, 2,5-diter butyl-1,4-benzoquinone (DBBQ) reported by Gao *et al.* raised the voltage of the mediated process above the onset potential of the direct O_2_ reduction at the cathode [9]. In the presence of DBBQ, O_2_ reduction did not follow the traditional LiO_2_ intermediate path and instead proceeded by forming an intermediate LiDBBQO_2_ complex in solutions (Fig. 2b). As a result, large Li_2_O_2_ with a toroidal morphology deposited via a solution-mediated mechanism, which significantly increased the discharge capacity by 80- to 100-fold and achieved better rate performance (Fig. 2c). Since then, the research enthusiasm has greatly been stimulated for RMs, especially the quinone derivatives, whose physicochemical properties (redox potential, solubility and electronic structure) can be modulated through interactions with the chemical environment. As demonstrated by Gray and co-workers, H_2_O can increase the thermodynamic stability of quinone monoanion and the associated O_2_ complex via hydrogen bonding. Therefore, in the presence of H_2_O, the discharge performance of DBBQ-mediated batteries was further improved (Fig. 2d) [19]. However, the existence of H_2_O may aggravate the deterioration of lithium metal anodes and even cause catastrophic fires or explosions. Accordingly, seeking more effective RMs is very urgent. Several biological anti-aging agents, such as coenzyme Q_10_ (CoQ_10_) [20] and Vitamin K2 [21], work in a fashion similar to DBBQ and exhibit praiseworthy results. In addition to organic molecules, inorganic redox couples, polyoxometalates such as ${\rm{\alpha - Si}}{{\rm{W}}_{{\rm{12}}}}{\rm{O}}_{{\rm{40}}}^{{\rm{4- }}}\!\! $, also demonstrate function as an ORR RM [22]. One key advantage of this system over other types of RMs is the expected stability of such clusters to reactive oxygenic species that can oxidatively decompose organic/organometallic species. The reaction characteristics are summarized in Table 1 [9,20–25] for some representative reductive RMs. Although most RMs significantly enhance the rate capability of Li–O_2_ batteries, their onset reduction potentials are only slightly more positive than that of oxygen. This means that they cannot satisfy the general standard for an ideal RM. Since the electronic properties of RMs can be tuned by engineering the molecular structures, Ye *et al.* introduced electron-withdrawing groups onto anthraquinone (AQ) moieties at different positions with different numbers, moving its reduction potential to a more positive region (Fig. 2e and f) [23]. As a result, the discharge performance of Li–O_2_ batteries was prominently improved (Fig. 2g). Furthermore, based on the systematic electrochemical performance, the correlation is well established between the reduction potentials of RMs and their catalytic performance.

**Table 1.** Summary of the main characteristics of representative ORR redox mediators in aprotic Li–O_2_ batteries.

RM	Electrolyte	Cathode	Current density	Discharge capacity
DBBQ [9]	10 mM DBBQ + 1.0 M LiTFSI in TEGDME	GDL	0.1 mA cm^–2^	436 ${\rm{mAh\ cm}}_{{\rm{BET}}}^{{\rm{-2}}}$
Q_10_ [20]	10 mM Q_10_ + 1.0 M LiTFSI in TEGDME	Super P	0.1 ${\rm{mA\ cm}}_{{\rm{areal}}}^{{\rm{-2}}}$	575 ${\rm{mAh\ cm}}_{{\rm{BET}}}^{{\rm{-2}}}$
BDTD [24]	20 mM BDTD + 1.0 M LiTFSI in TEGDME	CNT	0.1 ${\rm{mA\ cm}}_{{\rm{areal}}}^{{\rm{-2}}}$	4.7 mAh cm^–2^
VK2 [21]	10 mM VK2 + 1.0 M LiTFSI in DME	GDL	0.09 mA cm^–2^	3.6 mAh cm^–2^
${\rm{\alpha - Si}}{{\rm{W}}_{{\rm{12}}}}{\rm{O}}_{{\rm{40}}}^{{\rm{4-}}}$ [22]	50 mM ${\rm{\alpha - Si}}{{\rm{W}}_{{\rm{12}}}}{\rm{O}}_{{\rm{40}}}^{{\rm{4-}}}$+1.0 M LiTFSI in DMSO	Carbon cloth	0.1 uA cm^–2^	0.6 mAh cm^–2^
TTM [25]	Saturated TTM + 1.0 M LiOTF in TEGDME	Super P	0.1 mA cm^–2^	7.5 mAh cm^–2^
1,8 DNAQ [23]	10 mM 1,8 DNAQ + 0.5 M LiTFSI in TEGDME	Carbon paper	0.1 mA cm^–2^	2.25 mAh cm^–2^

GDL, gas diffusion layer; BDTD, benzo[1,2-*b*:4,5-*b*']dithiophene-4,8-dione; CNT, carbon nanotubes; VK2, vitamin K2; TTM, tris(2,4,6-trichlorophenyl)methyl.

### RMs for charge

Reducing the charge overpotential lies at the heart of the practical application of Li–O_2_ batteries. Due to the limited solid–solid contact, conventional solid catalysts seem difficult to obtain satisfactory performance. As a supplement, soluble RMs present the first step towards a new field and are rapidly attracting the attention of the Li–O_2_ battery research community. Since the Addison group patented to improve OER with RMs in 2011, numerous RMs have been developed, which can be categorized into organic, organometallic and inorganic ones [26].

#### Organic RMs

Organic RMs, such as tetrathiafulvalene (TTF) [8] and 2,2,6,6-tetramethylpiperidinyloxyl (TEMPO) [27,28], are a category of molecules with double bonds and/or aromaticity, which execute redox reactions via exchanging electrons at non-covalent structures.

**Figure 3.**
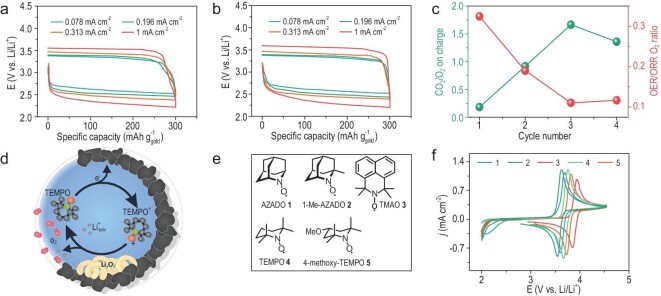
Cycling curves for the first (a) and 100th (b) cycle of Li–O_2_ batteries with 1 M LiClO_4_ in DMSO that contained 10 mM TTF at a nanoporous gold electrode under O_2_. The data are reproduced from Ref. [8]. (c) Ratios of O_2_ evolved on the charge to O_2_ consumed on discharge and CO_2_ to O_2_ evolved on charge as a function of the cycle number. Reproduced from Ref. [31]. (d) Proposed catalytic cycle for the electrochemical charging of Li–O_2_ batteries with TEMPO. Adapted with permission from Ref. [27] Copyright 2014, American Chemical Society. (e) Chemical structures of the investigated nitroxides. (f) CVs of 10 mM AZADO 1, 1-Me-AZADO 2, TMAO 3, TEMPO 4 and 4-methoxy-TEMPO 5 in 1 M LiTFSI/diglyme with a scan speed of 50 mV s^–1^, respectively. The data are reproduced from Ref. [32].

In 2013, TTF was reported as an effective RM in aprotic Li–O_2_ batteries [8]. Upon charge, TTF is directly oxidized to TTF^+^ at the electrode surface. Subsequently, TTF^+^ oxidizes solid Li_2_O_2_ products and then reverts to the initial neutral state. As a result, TTF effectively decomposes Li_2_O_2_ at a lower charge potential without side reactions. The round-trip efficiency was significantly improved and the cycle number was extended to as long as 100 (Fig. 3a and b). This extraordinary electrochemical performance inspired researchers to deeply explore the TTF-mediated OER process. By combining various analytic methods, Torres *et al.* disclosed that TTF^+^ acted as a ‘chemical scavenger’ by dissolving solid products deposited on the oxygen electrode, thus decreasing the charge overpotential and preventing the decomposition of electrolytes at high potentials [29]. However, the spectroscopic results of Ye and Qiao suggested that TTF may not be strongly involved in the oxidation of Li_2_O_2_, which will be discussed in detail below [30]. Such a conclusion was further emphasized by Yao *et al.*, who indicated that although TTF decreased the OER overpotential, it did not improve the coulombic efficiency (Fig. 3c) [31]. At the end of charge, massive CO_2_ was released, which means that most electrons transferred during charge were not used to oxidize Li_2_O_2_. These results obviously differed from those reported by the Bruce group [8] but the root reason has not been disclosed yet. Therefore, regarding the effect of RMs, it is necessary to conduct a variety of advanced characterizations to penetratingly expound the decomposition process of Li_2_O_2_.

Nitroxides, other prototypical organic RMs in Li–O_2_ batteries, are oxidized to anitroxides^+^ by losing an electron from the N–O group. TEMPO was introduced as a representative nitroxide RM by Janek and co-workers, which is schematically illustrated in Fig. 3d [27]. Although the oxidation potential of 3.74 V of TEMPO is higher than that of TTF, it can still serve as a suitable RM because parasitic reactions occurring at >4.0 V can be avoided successfully. As expected, the electrochemical performance of Li–O_2_ batteries was considerably ameliorated. Besides, electrochemical and physicochemical analyses demonstrated the high chemical/electrochemical stability of TEMPO, which guaranteed rapid diffusion kinetics for improving the rate capability. Generally, the chemical environment around the nitroxide group would influence their physicochemical properties, thus affecting the electrochemical performance of batteries. To gain insight into the structure–function relationship of nitroxide RMs, several nitroxide RMs with different chemical structures were systematically compared (Fig. 3e and f) [32]. The results showed that the steric protection of the nitroxide group played a critical role in their ability to reversibly donate and accept an electron. Besides, their redox potentials mainly depend on the chemical substituents next to the redox-active group. Therefore, introducing certain electron-donating R-groups (i.e. −N(CH_3_)_2_, −SCH_3_, −CH_3_, etc.) may contribute to lower charge potential and higher energy efficiency of Li–O_2_ batteries.

In addition to TTF and nitroxides, other organic RMs, such as 10-methyl-10H-phenothiazine (MPT) [33], tri dimethyl aminophelyl (TDPA) [34], and dimethylphenazine (DMPZ) [35], have also been widely employed in Li–O_2_ batteries, showing relatively low charge overpotential and long lifespan. Although most organic RMs feature good solubility in aprotic electrolytes, some with a large size present low mobility and ultimately slow kinetics. Flexible substitution of long hydrocarbon and branched hydrocarbon chains can regulate the solubility of molecules and be compatible with a variety of solvents. Additionally, through the functionality substitution, it is possible to manipulate the highest occupied molecular orbital and lowest unoccupied molecular orbital of RMs, thereby affecting their oxidation potentials, which is favorable to maximize the energy efficiency of Li–O_2_ batteries.

#### Organometallic RMs

**Figure 4.**
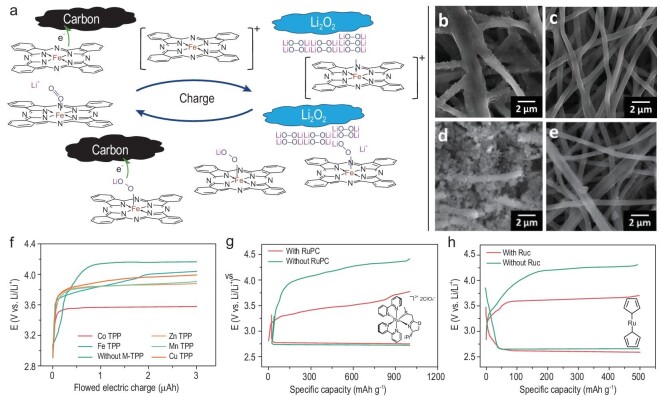
(a) The proposed OER catalytic mechanism of FePc in Li–O_2_ batteries. Scanning electron microscope images of the carbon fiber (CF) cathodes after discharge (b and d) and after the charge (c and e), without FePc catalyst (b and c) and with FePc catalyst (d and e). Adapted with permission from Ref. [36]. Copyright 2014 American Chemical Society. (f) Anodic chronopotentiograms obtained in the presence and absence of 1 mM metal complexes by use of the Li_2_O_2_-formed gass carbon (GC) electrode in Ar atmosphere, reproduced from Ref. [39]. (g) Voltage profiles of Li–O_2_ batteries without and with 0.05 M RuPC at a current density of 100 mA g^–1^ with a cut-off capacity of 1000 mAh g^–1^, reproduced from Ref. [38]. (h) Voltage profiles of Li–O_2_ batteries with 0.01 M Ruc in 0.1 M LiTFSI/tetraglyme at a current density of 0.1 mA cm^–2^ and capacity of 500 mAh g^–1^, reproduced from Ref. [40].

Organometallic RMs are composed of a central transition metal ion (M) stabilized by aromatic organic ligands, where M and organic ligands usually represent Co, Zn, Mn, Cu or Fe and bis(terpyridine), tetraphenylporphyrins (TPP) or phthalocyanine, respectively. Transition metal complexes are suitable OER RMs due to the fast outer-sphere electron transfer and the solubilizing/stabilizing properties conferred by organic ligands. Upon operation, the redox reactions are performed by changing the valence state of the active metal cations. In early 2014, Sun *et al.* first introduced iron-phthalocyanine (FePc) as an organometallic RM for Li–O_2_ batteries [36]. As shown in Fig. 4a, the Fe^III^/Fe^II^ couple in FePc with a redox potential of ∼3.65 V can chemically oxidize Li_2_O_2_. Notably, unlike organic RMs, most organometallic RMs can increase the discharge capacity by enhancing the solubility of oxygen and lithium oxide compounds. As a result, Li_2_O_2_ forms and decomposes without direct contact with the carbon electrode (Fig. 4b–e), which achieved a flat discharge plateau and a relatively steady charge end potential over 130 cycles. By contrast, the battery without FePc failed in the 21st cycle. Other molecules with similar catalysis were successively reported, such as cobalt bis(terpyridine) (Co(Terp)_2_) [31], Fe(heme) [37] and Ru(II) polypyridyl complex (RuPC) [38]. They not only reversibly accelerate Li_2_O_2_ formation and decomposition with a low overpotential but also effectively limit parasitic reactions. Because the catalytic activity of organometallic RMs highly depends on the metal ion, a series of metal macrocyclic complexes were investigated. The results showed that the charge potential of Li–O_2_ batteries with M–TPP increased in the following order: Co TPP < Zn TPP < Mn TPP < Cu TPP < Fe TPP (Fig. 4f) [39]. Besides, the structure of the organic compound/ligand greatly affects the electron density of center metal ions, and thus the redox potential of organometallic RMs. For instance, the RuPC-catalysed battery exhibited a charge potential at 3.50 V, which is 150 mV lower than the battery with ruthenocene (Ruc) (Fig. 4g and h) [38,40]. Similarly, the battery with FePc provided a distinct lower charge potential and higher coulombic efficiency than that of Fe(heme) [36,37]. Therefore, the properties of organometallic RMs can be flexibly modulated by modifying the molecular structure and/or replacing the metal cations. With that choice, the energy efficiency of Li–O_2_ batteries can be further optimized. However, such transition metal complexes with macrocyclic ligands usually exhibit slightly slow diffusion and poor solubility, which may depress the rate capability and power density of batteries, and flexible structure embellishment is expected to complement this shortcoming.

#### Inorganic RMs

**Figure 5.**
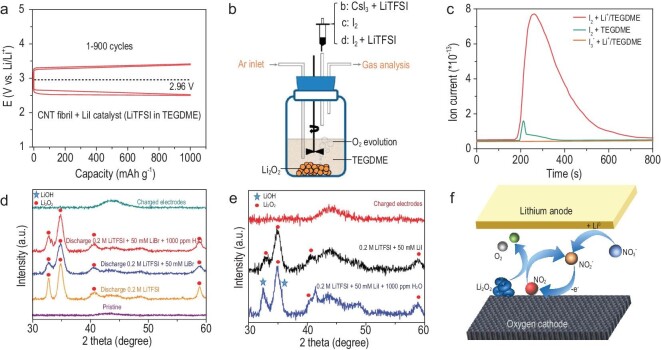
(a) Electrochemical cyclability of CNT fibril electrodes in the presence of a LiI catalyst, reproduced from Ref. [41]. (b) Schematic illustration of the mass spectrometer process (excess commercial Li_2_O_2_ powder and TEGDME are added in an argon-filled glass vial, then an equal amount of specified solution is injected into the glass vial, and the evolved gases are flowed into the gas analyser after stirring). Reprinted with permission from Ref. [44]. Copyright 2017 American Chemical Society. (c) Oxygen analysis was performed after the injection of 100 mM CsI_3_ + 1 M LiTFSI TEGDME, 100 mM I_2_ TEGDME, and 100 mM I_2_ + 1 M LiTFSI TEGDME solution into the argon-filled glass vial containing commercial Li_2_O_2_ powder and TEGDME, respectively, reproduced from Ref. [44]. X-ray diffraction patterns of carbon cathodes discharged to 2 V using solutions containing (d) LiBr or (e) LiI. The data are reproduced from Ref. [45]. (f) Schematic illustrations of the working mechanism of LiNO_3_.

Inorganic RMs contain halides, lithium nitrate (LiNO_3_) and some transition metal salts. In general, these agents promote Li_2_O_2_ decomposition by changing the oxidation state of active center ions. The operational mechanism of halides in Li–O_2_ batteries involves the following steps. First, the X^–^ ion is oxidized to ${\rm{X}}_{\rm{3}}^{{\rm{- }}}$, a polyhalogen anion. Then, ${\rm{X}}_{\rm{3}}^{{\rm{- }}}$ is converted to X_2_ and finally both ${\rm{X}}_{\rm{3}}^{{\rm{- }}}$ and X_2_ diffuse from the cathode surface to oxidize Li_2_O_2_ products. Lithium iodide (LiI), a controversial RM, was first reported by Lim *et al.* in 2014 [41]. Combined with a hierarchical nanoporous air electrode, the battery achieved a significantly reduced overpotential (0.25 V) and high cyclic stability (>900 cycles) (Fig. 5a). Notably, the polarization did not drastically increase, even when the current density was 30 times higher. Although LiI did promote the battery performance in many reports, its catalytic mechanism is still under debate, which mainly focused on the discharge products and specific catalytic active species. In early 2015, Gray *et al.* found that in the presence of H_2_O, LiI could affect the chemical composition and morphology of discharge products [42]. However, further studies disclosed that even without H_2_O, when the LiI concentration was high, the salt promoted the formation of LiOH [43]. Another controversy focused on the active species that catalyses the decomposition of Li_2_O_2_. Initially, much evidence showed that the I^–^/${\rm{I}}_{\rm{3}}^{{\rm{- }}}$ redox couple with a lower redox potential is responsible for the chemical decomposition of Li_2_O_2_. Nevertheless, the chemical simulation performed by Cui *et al.* suggested that the effective oxidation state of I^–^ for oxidizing Li_2_O_2_ was I_2_ species rather than ${\rm{I}}_{\rm{3}}^{{\rm{- }}}$ (Fig. 5b and c) [44]. This discrepancy may originate from the different fundamental natures (crystalline, distribution and morphology) of the electrochemically generated Li_2_O_2_ and the commercial bulk Li_2_O_2_. In other words, the results gained from the prefilled electrode cannot sufficiently explain the real charge process. Besides, the impurities and surface contaminations are different in the commercial Li_2_O_2_ powders and the electrochemically formed Li_2_O_2_, which also confuse the assessment of catalytic effects of LiI. Therefore, when evaluating the catalytic ability of RMs, it is important to reflect on the actual battery situation to avoid misunderstanding.

Compared with LiI, lithium bromide (LiBr) has a similar operation mechanism but a high redox potential of 3.5 V, which can suppress charging side reactions at a high potential. Different from ${\rm{I}}_{\rm{3}}^{{\rm{- }}}$, ${\rm{Br}}_{\rm{3}}^{{\rm{- }}}$ is not oxidized to Br_2_ in the usual working potential range and thus a clearer working mechanism is known. In addition, LiBr is more stable than LiI as it is less prone to nucleophilic attack by ORR intermediates. As shown in Fig. 5d and e, LiOH forms in LiI-assisted batteries. However, the discharge products of LiBr-assisted batteries are mainly Li_2_O_2_, even with different solvents and water contaminations [45]. These results indicate that the redox potential of RMs is not the only criterion for judging the catalytic effect, and the compatibility of RMs and battery environment also play a vital role and cannot be neglected.

LiNO_3_, an electrolyte additive commonly used as the solid–electrolyte interface (SEI) stabilizer for anodes, has been demonstrated to mediate Li_2_O_2_ oxidation. Unlike halides, the redox couple in LiNO_3_ is the anion group NO_2_^–^/NO_2_, which is generated by LiNO_3_ reduced at Li anodes. Then, NO_2_^–^ migrates to the cathode and is oxidized to NO_2_ gas at ∼3.6 V and finally NO_2_ gas chemically oxidizes Li_2_O_2_ (Fig. 5f). Generally, NO_2_ gas is inclined to vaporize in the open structure and cannot sustain NO_2_^–^/NO_2_ redox reactions. However, recent research has suggested that LiNO_2_ would be spontaneously oxidized by O_2_ to LiNO_3_. Furthermore, the conversion to NO_3_^–^ occurs at a much higher rate than the vaporization of NO_2_. Therefore, NO_2_^–^ can be regenerated and reused in the next cycle [46]. However, LiNO_3_ only works when it comes into contact with Li metal, which considerably limits its application because sometimes Li metal must be separated from the electrolyte to avoid dendrite growth.

### Dual RMs and bifunctional RMs

To promote the practicality of Li–O_2_ batteries, a large discharge capacity and a small overpotential must be simultaneously achieved. Nevertheless, most RMs can only handle one of these two issues. Therefore, dual RMs or bifunctional RMs are undoubtedly worthy for Li–O_2_ batteries.

**Figure 6.**
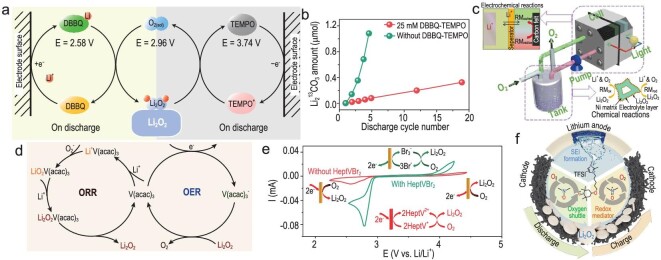
(a) Schematics illustration of the OER and ORR processes in Li–O_2_ batteries with DBBQ and TEMPO. (b) Amounts of Li_2_^13^CO_3_ in the ^13^C-carbon cathodes at the end of discharge on each cycle, reproduced from Ref. [47]. (c) Configuration of the redox flow Li–O_2_ battery with a pair of RMs. Adapted with permission from Ref. [49]. Copyright 2015 Royal Society of Chemistry. (d) Schematic illustration of ORR and OER in the cell with V(acac)_3_. Adapted with permission from Ref. [51]. Copyright 2019 Wiley-VCH Verlag GmbH & Co. KGaA, Weinheim. (e) CV curves and schematic reactions for ORR and OER with and without HeptVBr_2_, reproduced from Ref. [54]. (f) Schematic illustration of the IL-TEMPO facilitating the performance of Li–O_2_ batteries. Adapted with permission from Ref. [57]. Copyright 2019 Nature publishing group.

The combination of dual RMs is supposed to exhibit a synergistic effect to facilitate both ORR and OER processes. The attempt was typically performed by the Bruce group, who reported a Li–O_2_ battery assisted by dual RMs, DBBQ and TEMPO (Fig. 6a) [47]. Specifically, the corrosion of carbon electrodes, a major barrier to the progress of Li–O_2_ batteries, was significantly mitigated by forming/decomposing Li_2_O_2_ in solutions and avoiding high charge potentials (Fig. 6b). In this way, a Li–O_2_ battery was realized with larger discharge capacity, lower charge overpotential and higher reversibility. Although the dual RMs-assisted battery performance is no longer discounted by the sluggish ORR and ORR kinetics, the limited practical capacity and rate performance are still subjected to the narrow O_2_ mass transport. To conquer this obstacle, a dual RM battery with a ‘liquid Teflon’-type binary perfluorocarbon was deliberately designed, which demonstrated an enhanced discharge capacity of 6 mAh cm^–2^ at a current density of 50 μA cm^−2^ [48]. Furthermore, based on the ‘redox targeting’ concept, a novel rechargeable redox flow Li–O_2_ battery was developed (Fig. 6c) [49]. In this case, electrolytes and O_2_ are easily circulated by a peristaltic pump, and the formation and decomposition of Li_2_O_2_ proceeded in a separate gas diffusion tank. Consequently, the batteries obviated surface passivation and presented high energy density and good rechargeability. However, employing multiple RMs inevitably aggravates the complexity of Li–O_2_ batteries. Thus, researchers are urgently eager for bifunctional RMs that can synchronously address OER and ORR problems.

According to the ORR mechanism, current bifunctional RMs can be divided into two categories. One is to tune the ORR process by binding Li^+^ or superoxide species (O_2_^–^ and LiO_2_) and reducing the charge potential by redox shuttle [50]. For example, the recently reported vanadium(III) acetylacetonate (V(acac)_3_) integrates with the superoxide intermediate, thus accelerating O_2_ reduction and suppressing undesired parasitic reactions [51]. During charge, V(acac)_3_ acts as an electron carrier to chemically oxidize Li_2_O_2_ (Fig. 6d). Most organometallic compounds belong to this category. Besides, some molecules with special functional groups can also realize bifunctional catalysis. For instance, the dipolar N–O bond in 2-phenyl-4,4,5,5-tetramethylimidazoline-1-oxyl-3-oxide (PTIO) increased the level of oxygen species in solutions, thereby improving the discharge performance. Meanwhile, the redox couple of PTIO^+^/PTIO enables the decomposition of Li_2_O_2_ with a lower charge plateau [52]. In the actual battery operation, nevertheless, parasitic products are inevitable due to the decomposition of electrolytes, which will hinder the function of the RMs. Therefore, Zhang *et al.* fabricated a new RM 2,5-di-tert-butyl-1,4-dimethoxybenzene (DBDMB) with a redox potential at 4.20 V, which not only enabled the solution growth of Li_2_O_2_ by capturing the reactive O_2_^–^ but also efficiently oxidized Li_2_O_2_ products and parasitic products [53]. Note that this type of RMs is, however, unable to stop surface passivation entirely as the direct electrochemical reduction of O_2_ still occurs.

Other bifunctional RMs facilitate ORR and OER through the EC mechanism. For instance, 1,1^′^-diheptyl-4,4^′^-bipyridinium (heptyl viologen) dibromide (HeptVBr_2_), with two redox couples of ${\rm{Hept}}{{\rm{V}}^ + }/{\rm{Hept}}{{\rm{V}}^{2 + }}$${\rm{and}}$${\rm{B}}{{\rm{r}}^ - }/{\rm{Br}}_{\rm{3}}^{{\rm{- }}}$, promotes the formation/decomposition of Li_2_O_2_ concurrently (Fig. 6e) [54]. Specifically, reduced viologen-based species combine with O_2_ and Li^+^ to generate Li_2_O_2_ upon discharge $( {{\rm{L}}{{\rm{i}}^{\rm{ + }}}{\rm{\ + \ Hept}}{{\rm{V}}^{\rm{ + }}}{\rm{\ + \ O}}_{\rm{2}}^{{\rm{- }}}{\rm{\ }} \to {\rm{\ L}}{{\rm{i}}_{\rm{2}}}{{\rm{O}}_{\rm{2}}}{\rm{\ + \ Hept}}{{\rm{V}}^{{\rm{2 + }}}}} )$. Upon charge, Br^–^ is electrochemically oxidized to ${\rm{Br}}_{\rm{3}}^{{\rm{- }}}$ and then chemically oxidizes Li_2_O_2_. Several inorganic salts, such as MoCl_5_ [55] and CuCl_2_ [56], have also been demonstrated to regulate the oxygen electrochemistry via their different redox couples. In addition to the ingenious molecular selection, rationally artificializing novel molecules was identified to be an effective way to achieve multiple effects. Wang *et al.* fabricated a TEMPO-grafted ionic liquid (IL) as a multifunctional agent for Li–O_2_ batteries (Fig. 6f) [57]. Besides the redox shuttle endowed by the n-/p-doping property, a stable SEI would form. The combination of these unique properties even allows batteries to be operated in the air atmosphere, which makes it potentially suitable for future practical applications. Despite this, research on bifunctional RMs has only started in the last several years, which will be an important step in the realization of practical Li–O_2_ batteries.

## CHALLENGES

To be objective, although RMs provide a new prospect for Li–O_2_ batteries, the problems raised cannot be ignored: (i) matching of RMs with battery components (electrode materials, solvents, salts, etc.) is not clear; (ii) redox shuttle of RMs leads to the corrosion of Li anodes and loss of the catalytic activity of RMs; (iii) some organic RMs may be subject to similar decomposition to the electrolyte or carbon; (iv) there is no consensus on the factors on the dynamics of the reaction between RMs and reactants.

### Choice of RMs

As mentioned above, the redox potential of RMs greatly defines the operational potential of batteries and thus the energy efficiency. Despite an inherent characteristic, the actual redox potential of RMs in batteries could be affected by battery components. Besides, the transport pathway of RMs in electrolytes, which is usually impacted by cathode architectures, is essential for high-rate performance. Consequently, it is crucial to understand the interplay between cathodes, electrolytes (solvents and salts) and RMs employed.

**Figure 7.**
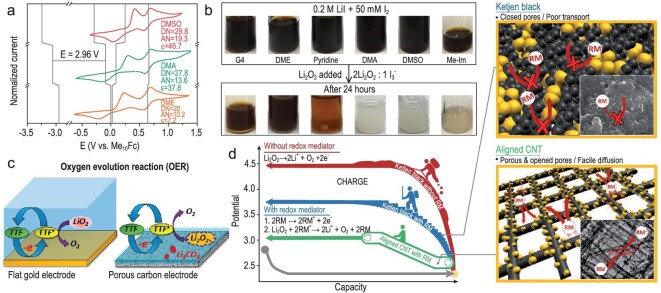
Solvent-dependent redox potentials of ${\rm{I}}_{\rm{3}}^{\rm{-}}$/I^–^. (a) CVs of solutions of 0.5 M LiTFSI + 10 mM LiI collected at 100 mV s^–1^ under an Ar environment in each of the considered solvents, reproduced from Ref. [58]. (b) Solvent-dependent reactions between ${\rm{I}}_{\rm{3}}^{\rm{-}}$/I^–^ and I_2_/${\rm{}}_{\rm{3}}^{\rm{-}}$ and Li_2_O_2_. Reprinted with permission from Ref. [58]. Copyright 2019 Elsevier. (c) Schematic illustration of OER in the cell containing TTF with different oxygen cathodes, reproduced from Ref. [30]. (d) Schematic illustration of the role of RMs in the Li–O_2_ battery with different carbon electrodes. Reprinted with permission from Ref. [41]. Copyright 2014 Wiley-VCH Verlag GmbH & Co. KGaA, Weinheim.

From the thermodynamic analysis, the equilibrium potential is a key parameter for choosing the RMs, which is dependent on the Gibbs free energy change between the reduced and the oxidized species in a particular electrolyte. As the Gibbs free energy of Li^+^ in electrolytes can be tuned by designing an appropriate electrolyte, the redox potential of soluble RMs will also be affected by electrolytes. For example, Shao-Horn and co-workers disclosed that the activity of LiI was greatly affected by electrolytes (Fig. 7a and b) [58]. The solvents with stronger solvation of I^–^ such as *N,N*-dimethylacetamide (DMA), dimethyl sulfoxide (DMSO) and 1-methylimidazole (Me-Im) drastically enhanced the oxidizing power of ${\rm{I}}_{\rm{3}}^{{\rm{- }}}$, which allowed more Li_2_O_2_ to decompose. This solvent-dependent oxidizing power of RMs was consistent with the results of Hung *et al.*, who found that the rate of O_2_ evolution associated with the reaction kinetics between ${\rm{I}}_{\rm{3}}^{{\rm{- }}}$ and Li_2_O_2_ greatly depended on the electrolyte solvent [59]. Furthermore, Pande and Viswanathan suggested that if the RM size is larger, the solvent will have less influence [60]. As another key component in electrolytes, salts also alter the activity of RMs, especially the concentration, which is similar to the equilibrium potential of Li/Li^+^ varying with the salt concentration [61]. Besides, the concentration of RMs also influences the battery performance. As reported previously, the high concentrations of LiI in ether solutions facilitated the side reaction generating a primary product LiOH [43]. Therefore, the electrolyte-dependent activity of RMs indicates that the component electrolyte deserves to be explored thoroughly, including the type of solvents and the concentration of salts and RMs. Besides, the stability of electrolytes should also be taken into consideration.

In addition, electrode materials and interface engineering could also influence the catalytic power of RMs. For example, Ketjen Black (KB) carbon cathodes severely damage the stability of DBBQ, whereas the non-carbon porous antimony tin oxide cathode showed improved stability against RM degradation, emphasizing that the stability of RMs can be controlled by proper electrode materials [62]. Specifically, the surface characteristics of electrodes could influence the catalytic mechanism of RMs. As reported by Ye and Qiao, the functionality of TTF strongly depended on the electrode materials and morphologies (Fig. 7c) [30]. When the gold electrode was used, the TTF^+^ was predominantly consumed by the oxidative decomposition of LiO_2_ instead of Li_2_O_2_. When porous carbon electrodes were used, although the decomposition of Li_2_O_2_ was promoted, the interaction of TTF^+^ moieties with carbon electrodes seemed to badly affect the stability. Besides, the accessibility of RMs to Li_2_O_2_ products—that is, the cathode structure—will influence the electron transfer. For example, compared with KB, the hierarchically aligned porous electrode provided a more facile diffusion path for RMs in electrolytes [41]. As a result, a highly efficient, rechargeable Li–O_2_ battery was realized (Fig. 7d). Moreover, the crystal facets of Li_2_O_2_ could influence the reactivity of RMs. As demonstrated recently, the increase in potentials led to the exposure of new Li_2_O_2_ facets that react with RMs, which significantly enhanced the oxidation of Li_2_O_2_ by ${\rm{Br}}_{\rm{3}}^{{\rm{- }}}$ [63]. Therefore, future research can focus on improving the RM reaction rate by regulating product characteristics. Previous reports revealed that the formation of defective or amorphous Li_2_O_2_ can be induced with electrocatalysts and well-designed porous cathodes [64,65]. These strategies are expected to be effective in improving the reactivity between RMs and Li_2_O_2_. Overall, the key challenge for future Li–O_2_ batteries is synergistically to combine diverse modulation strategies for overall performance enhancement.

### Redox shuttle of RMs

Due to the soluble nature of RMs, they can freely diffuse/migrate between the cathode and lithium anode, which is termed as the ‘shuttle effect’. Although originally developed for overcharge protection in Li-ion batteries, the shuttle effect is not expected in Li–O_2_ batteries, as it usually induces the deterioration of Li anodes and the functional depletion of RMs [66,67]. Besides, some undesirable species originating from Li-metal corrosion may dissolve into the electrolyte and then migrate to the cathode, which is detrimental to the stability of Li–O_2_ batteries. Attempts have been made to suppress intractable redox shuttle, which can be divided into three categories (modifying separators, designing cathodes and protecting anodes) according to the functional position.

**Figure 8.**
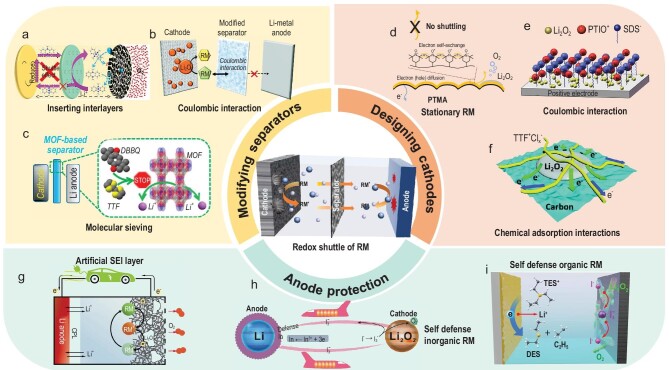
Strategies to inhibit redox shuttle of RMs. (a) Schematic illustration of the role of Li^+^–Nafion separator for preventing DMPZ-induced shuttle effect during charge [68]. (b) Schematic illustration of the working mechanisms of Li–O_2_ batteries with the modified separator [35]. (c) Schematic images of a MOF-based separator, acting as a sieve to inhibit the shuttle of RM molecules [72]. (d) Schematic of working processes of PTMA [74]. (e) Mechanism of particle distribution on the electrode surface during charging with SDS [52]. (f) Schematic illustration of the mechanism of TTF^+^Cl_x_ facilitating the decomposition of Li_2_O_2_ [75]. (g) Schematic illustration of the CPL-coated Li electrode in Li–O_2_ batteries, which prevents the reaction between the redox mediator and the Li-metal electrode [76]. Schematic illustration of a self-defense redox mediator of (h) InI_3_ [77] and (i) TESI [78] in Li–O_2_ batteries. Reprinted with permission from: (a) Ref. [68]. Copyright 2018 The Royal Society of Chemistry; (b) Ref. [35]. Copyright 2017 Wiley-VCH Verlag GmbH & Co. KGaA, Weinheim; (c) Ref. [72]. Copyright 2018 American Chemical Society; (d) Ref. [74]. Copyright 2020 Wiley-VCH Verlag GmbH & Co. KGaA, Weinheim; (e) Ref. [52]. Copyright 2017 American Chemical Society; (f) Ref. [75]. Copyright 2017 Wiley-VCH Verlag GmbH & Co. KGaA, Weinheim; (g) Ref. [76]. Copyright 2016 The Royal Society of Chemistry; (h) Ref. [77]. Copyright 2019 Nature publishing group; (i) Ref. [78]. Copyright 2016 Wiley-VCH Verlag GmbH & Co. KGaA, Weinheim.

Inserting interlayers is the most intuitive approach to physically prevent the RMs from reaching the anode. Nafion with high ion selectivity and Li-ion transfer capability is regarded as a worthwhile material to decorate conventional separators. As a prototype of such an approach, Zhou and co-workers originally proposed to prohibit RMs crossover towards Li-metal anodes with a single ion-conducting Li^+^–Nafion separator (Fig. 8a). In their study, the self-discharge and shuttle problems of RMs are effectively avoided. The battery kept low charge overpotentials of 0.24 V during the long-term cycling [68]. In a parallel effort, a NASICON (sodium super ionic conductor)-type Li_1+x+y_Al_x_(Ti, Ge)_2__–__x_Si_y_P_3__–__y_O_12_ (LATGP) ceramic solid electrolyte was employed [69]. In addition to inhibiting the shuttle of TEMPO, the LATGP membrane also protected the cathode from the chemical attack of soluble components in the anode SEI such as carbonate, acetate and formate. Employing solid electrolytes could increase the mass of batteries and lower the mobility of Li^+^, and thus severely reduce the energy density and rate capability of batteries. Alternatively, functionally modifying the separator gained considerable attention. The modification principle is to block the diffusion pathway of RMs through a physical barrier or coulombic interactions [70,71]. As shown in Fig. 8b, the fabrication of a commercial glass fiber separator coated with a negatively charged polymer mitigated the migration of DMPZ through coulombic interactions between the decorated separator and the oxidized RMs [35]. Nevertheless, anchoring RMs with electrostatic interactions is less effective in improving cycling stability due to the weak binding force. By contrast, a modified separator with a narrow pore-size window—that is, an RM molecular sieve—has an overwhelming advantage in overall electrochemical performance. For example, a metal–organic framework (MOF)-based separator with a size window of ∼6.9–9 Å effectively inhibited the RMs migration while keeping the Li^+^ permeation (Fig. 8c) [72]. In this case, the Li–O_2_ battery maximized the advantages of the dual mediator strategy, revealing a prolonged cycled life (100 cycles, 5000 mAh g^–1^) at a high current rate (1000 mA g^–1^). However, the weight proportion of modified separators is the biggest concern because it decreases the energy density of batteries.

Another strategy to inhibit the shuttle effect is restricting the movement of RMs by elaborately designing cathodes. The simplest method is directly immobilizing the RMs to the cathode with a linker. However, the participation of poorly conductive adhesives will inevitably lead to underused RMs. By comparison, Peng *et al.* electrochemically fabricated a thin conductive polymer film of poly-anthraquinone (PAQ) [73]. While ensuring the charge transfer, the shuttle of AQ was commendably suppressed. Therefore, it showed comparable rate capability to the AQ-assisted battery. Furthermore, Kang and co-workers creatively demonstrated that polymer-based RMs, poly(2,2,6,6-tetramethyl-1-piperidinyloxy-4-yl methacrylate) (PTMA), decoupled the redox property of RMs and shuttle effect by anchoring the RMs at the electrode surface [74] (Fig. 8d). Specifically, the physical migration of RMs was replaced by charge transfer along polymer chains. Moreover, the functional modification of cathodes is capable of suppressing the shuttle effect of RMs by physical/chemical adsorption. As shown in Fig. 8e, the non-electroactive surfactant (sodium dodecyl sulfate, SDS) could be adsorbed *in situ* on the hydrophobic carbon surface and form a stable anionic layer upon charge, thereby admirably restraining the PTIO diffusion through electrostatic attraction [52]. However, as mentioned above, the electrostatic adsorption is very weak compared with chemical adsorption; therefore, it is difficult to obtain satisfactory electrochemical performance, especially in long-cycle tests. As an improvement, Wang *et al.* introduced lithium chloride (LiCl) to the electrolyte, forming an electronic conductor solid organic compound (TTF^+^Cl_x_) covering the electrode surface (Fig. 8f) [75]. This conductive compound not only restricted TTF^+^ movement around the cathode but also provided efficient electron-transport pathways. Overall, engineering cathodes are promising for mitigating the shuttle effect and improve the performance of Li–O_2_ batteries.

Although numerous methods have positive effects on suppressing the shuttle effects, some RMs can still reach the Li anode side. Therefore, protecting the Li anode from reacting with RMs is the last chance to conquer the redox shuttle. An *exsitu* artificial SEI layer, a stable thin layer on the Li anode surface, can restrain the growth of Li dendrites and the redox shuttle of RMs, thereby significantly improving the decomposition efficiency of Li_2_O_2_ and the battery cycle (Fig. 8g) [76]. Due to the pretreatment property, the artificial layer provides more options to manipulate their component, morphology and structure. However, the physically coated layers may affect Li-ion transportation and increase preparation costs in some cases. In contrast, it is more feasible to form an *insitu* passivation layer on the Li anode surface. For example, the In^3+^ cation in indium tri-iodide (InI_3_) can electrodeposit onto the Li anode before Li^+^ during charging, spontaneously forming a Li–In alloy-containing SEI layer (Fig. 8h) [77]. With the Li–In alloy-based layer, the chemical reduction of I_3_^–^ at Li anodes and Li dendrites was effectively impeded. Simultaneously, I^–^/${\rm{I}}_{\rm{3}}^{{\rm{- }}}$ still acted as a redox couple to chemically decompose Li_2_O_2_. This dexterous strategy of killing two birds with one stone opens up a new avenue to increase the efficiency of RMs. Similarly, some organic halides containing special functional groups also acted as both charge carriers and SEI-forming agents for Li–O_2_ batteries [78,79], as demonstrated in Fig. 8i. However, these naturally forming SEI films are usually unstable and vulnerable during repeated cycles, which may be the main obstacle of RMs-based Li–O_2_ batteries.

### Stability of RMs

To make RMs-assisted Li–O_2_ batteries cycle stably, RMs must be fully utilized over the repeated cycles without losing the efficiency or content. Although the mobile characteristic endows RMs with a desirable catalytic effect, it also causes RMs to come into direct contact with every component and chemical species in batteries, leading to underlying decomposition. Degradation of RMs would be even more detrimental than the electrolyte and cathode although the concentration of RMs is low.

**Figure 9.**
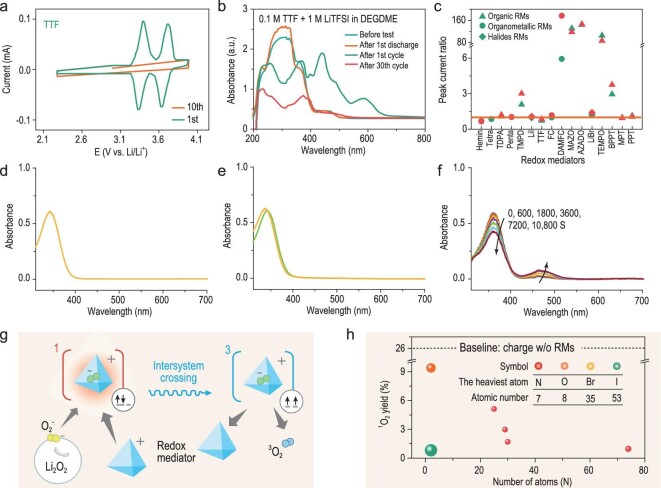
(a) CVs of bi-compartment batteries for the 1st and 10th cycles with 1 M LiTFSI/DEGDME (diethylene glycol dimethyl ether) solutions containing 0.02 M TTF under Ar atmospheres (scan rate: 0.1 mV s^−1^, voltage range: 2.3–4.0 V); (b) UV-vis solution spectra of 1 M LiTFSI in DEGDME electrolyte containing 0.1 M TTF at different conditions (before the electrochemical testing, after first discharge, after first cycle and after 30 CV cycles in bi-compartment cells under O_2_ atmosphere). The data are reproduced from Ref. [81]. (c) The peak current ratios, |I_p, a_/I_p, c_| of tested RMs in DMSO solvent, reproduced from Ref. [86]. UV-vis spectra of 60 μM DMPZ against oxygen species in 0.1 M LiTFSI/TEGDME electrolyte before and after exposure to (d) O_2_, (e) KO_2_ and (f) ^1^O_2_. Reproduced from Ref. [87]. (g) Schematic illustration of suppressing ^1^O_2_ through intersystem crossing (ISC, a radiationless transition between two electronic states with different spin multiplicities) via a RM. Reprinted with permission from Ref. [15]. Copyright 2020 American Chemical Society. (h) Comparison of ^1^O_2_ yields in charging with various RMs, together with the number of atoms and the atomic number of the heaviest atom in the mediators, reproduced from Ref. [15].

As mentioned above, much attention has focused on the redox shuttle of RMs, which was generally considered to be the main reason for the decrease in RM activity in Li–O_2_ batteries. Nevertheless, even if the Li anode was completely isolated from the cathode side, the cycle of RMs-assisted batteries was still very limited. This phenomenon reminds us that the stability of RMs in the harsh electrochemical environment needs to be further investigated. Chen’s group studied the stability of TTF by CV [80]. Almost consistent CV curves during 1–20 cycles under O_2_ atmosphere implied the RM stability and ignorable side reactions. However, only CV assessment without rigid spectroscopic evidence would miss some possible undesired reactions. To provide a realistic view on the stability of RMs, Sun *et al.* designed a bi-compartment cell and performed electrochemical and spectroscopic analyses [81]. Unexpectedly, no obvious redox peaks of TTF were observed in CV curves after 10 cycles (Fig. 9a). The changes in ultraviolet-visible (UV-vis) spectra of solutions after cycling were fully in line with the electrochemical data, which should be attributed to the deactivation of TTF (Fig. 9b). Similar results were obtained by TEMPO and DMPZ, indicating that even under Ar atmosphere and not in contact with Li anodes, RMs still suffered from the intrinsic decomposition. Although narrowing the operational potential may improve the stability of RMs to some extent, it is not suitable for competed ORR and OER cycling. Fortunately, the physiochemistry properties of RMs can be tuned by appropriate structural modifications, such as replacing or chemically modifying the functional group involved in the deterioration reactions. Especially, organometallic RMs, whose central metal ion is surrounded by cyclic organic ligands, can be better protected from chemical attack via steric protection of side groups. However, due to the large size, this strategy may cause new issues relating to the low mobility and slow kinetics of RMs.

In addition to the intrinsic stability, RMs also undergo further examination in a harsh oxygen environment. Previous studies suggested that almost all non-aqueous solvents used to date are not stable towards the oxygen reduction species: O_2_^–^, LiO_2_ and Li_2_O_2_ [82]. Therefore, RMs, especially organic RMs with C–H bonds next to O or N atoms, may be prone to being attacked by the aggressive oxygen species in Li–O_2_ batteries [83]. In the presence of Li^+^ ions, the nucleophilic attack is further exacerbated, which will trigger aggravated parasitic reactions that jeopardize the cycle life of batteries [84,85]. Accordingly, screening out stable RMs has extraordinary significance for the development of Li–O_2_ batteries. After comprehensively investigating the stability of 20 RMs in Li–O_2_ batteries via CV and galvanostatic cycling tests, Khojin *et al.* found that the stability of RMs followed the order of halides > organics > organometallics (Fig. 9c) [86]. Density functional theory computations suggested that organic RMs are vulnerable to ^1^O_2_ released from the decomposition of Li_2_O_2_. Although halide RMs (LiI and LiBr) are not susceptible to ^1^O_2_, they are nucleophiles and can induce electrolyte degradation [43]. Besides, it would cause other parasitic reactions with trace H_2_O in batteries, forming by-products such as LiOH and LiOOH at the expense of Li_2_O_2_. As researchers have verified that ^1^O_2_ is the culprit of parasitic reactions, Sun *et al.* assessed the reactivity of organic RMs towards dissolved O_2_, O_2_^–^, Li_2_O_2_ and ^1^O_2_ with precise quantitative analyses. They disclosed that the deactivation of RMs in Li–O_2_ batteries was predominantly caused by the attack of ^1^O_2_, as presented in the UV-vis spectroscopy of Fig. 9d–f. Reactions with superoxides, previously assumed to mainly trigger their degradation, peroxides and dioxygen, were orders of magnitude slower in comparison. Besides, due to the electrophilic nature of ^1^O_2_, the reduced RMs were particularly more vulnerable to ^1^O_2_ than the oxidized form [87]. These results encourage researchers to carefully design RMs sufficiently stable for long-term operation. Suppressing the ^1^O_2_ formation by quenchers is expected to alleviate the loss of RMs. For example, 1,4-diazabicyclo[2.2.2]octane (DABCO), the most efficient quencher used in Li–O_2_ batteries, was proposed to protect DMPZ from the attack by ^1^O_2_ and achieved satisfactory performance [88]. However, DABCO is not sufficient to eliminate ^1^O_2_ due to the narrow stability voltage window and limited quenching rate constant. By contrast, Lu *et al.* revealed the universal effect of RMs in suppressing ^1^O_2_ during the charge of Li–O_2_ batteries (Fig. 9g) [15]. The investigated RMs displayed up to three orders of magnitude higher ^1^O_2_ suppression efficiency compared with DABCO. They also found that RMs with more atoms or heavy atoms have stronger ^1^O_2_ suppression ability (Fig. 9h), which is consistent with intersystem crossing promotion by enhancing spin–vibronic coupling and spin–orbit coupling. These results provide rational guidelines to design RMs for efficient and reversible Li–O_2_ batteries.

### Kinetics of Li_2_O_2_ oxidation

**Figure 10.**
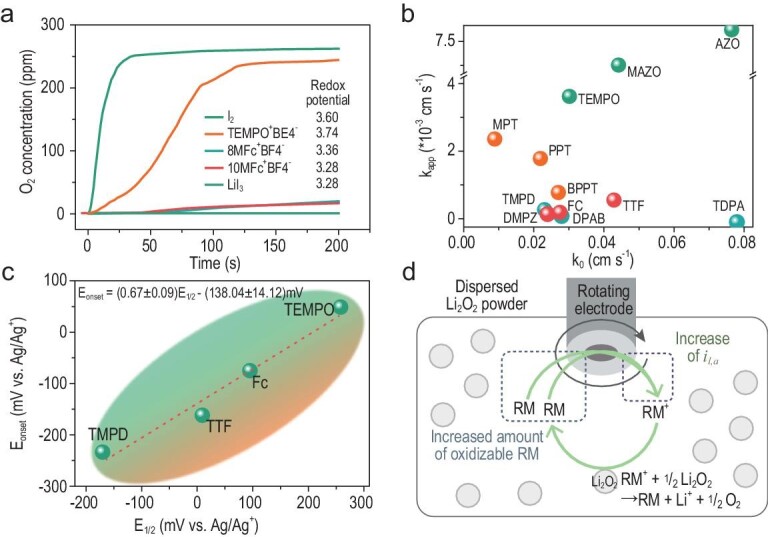
(a) Oxygen concentration after Li_2_O_2_ was added into TEGDME solvent containing different oxidized RMs based on the data reproduced from Ref. [59]. (b) Dependence of the apparent rate constant, *k*_app_, on the heterogeneous electron-transfer rate constant, *k*_0_, of the RMs. The data are reproduced from Ref. [89]. (c) The plot of *E*_onset_ as a function of *E*_1/2_ for the different studied RMs, reproduced from Ref. [83]. (d) Schematic displaying the increase in the oxidative current by the EC mechanism. Reprinted with permission from Ref. [91]. Copyright 2019 The Royal Society of Chemistry.

RMs improve the power capability of Li–O_2_ batteries by replacing the sluggish discharge/charge process with a facile redox-mediated reaction. The rapid and sufficient oxidation of Li_2_O_2_ by RMs is essential for the high-rate capability and superior reversibility of Li–O_2_ batteries. It is often assumed that RMs with high redox potentials have fast kinetics for the oxidation of Li_2_O_2_; however, this is not necessarily so. As suggested by measuring the oxygen evolution rate, an indication of the reaction kinetics between RMs and Li_2_O_2_, there was no definite relationship between the oxidation rate of Li_2_O_2_ and the redox potential of RMs (Fig. 10a) [59]. Such a conclusion aroused great interest in investigating the oxidation kinetics of Li_2_O_2_ with RMs and more in-depth studies on the reaction chemistry were conducted. With scanning electrochemical microscopy, the Bruce group indicated that there was no correlation between k_app_ (the apparent reaction constant of Li_2_O_2_ oxidation by RM^+^) and k_0_ (the reaction constant of RM oxidation by heterogeneous electron transfer), let alone the redox potential of RMs (Fig. 10b) [89]. They claimed that the electron transfer between RM^+^ and Li_2_O_2_ was based on an inner-sphere reaction, where the adsorption between them played a critical role in the reaction rate. Consequently, the steric structure of RMs greatly influences the oxidation kinetics of Li_2_O_2_. When the redox center of RMs is surrounded by bulky groups, the oxidation rate of Li_2_O_2_ will decrease. This conclusion was consistent with the results [90]; compared with TEMPO, the 2-azaadamantane-N-oxyl (AZADO) molecule with a smaller steric effect and higher electron-donating power exhibited higher catalytic activity and thus lowered charging overpotential. Nevertheless, a different viewpoint on the electron transfer of RMs-assisted OER process was presented by Baltruschat *et al.* By using a new thin-layer cell-related differential electrochemical mass spectrometry (DEMS), they established a linear relationship between *E*_onset_ (the onset potential of oxygen evolution) and *E*_1/2_ (the half-wave potential of RM redox) (Fig. 10c). It suggested that the Li_2_O_2_ oxidation by RM^+^ was an outer-sphere reaction that can be explained by Marcus theory [83]. However, the *E*_onset_ cannot signify the entire OER process; therefore, it is flawed in reflecting the reaction kinetics. Subsequently, Kang *et al.* comparatively studied the kinetics of RMs-mediated Li_2_O_2_ decomposition by probing linear sweep voltammetry (LSV) with a rotating disk electrode. The schematic diagram of the mechanism is shown in Fig. 10d. When excess Li_2_O_2_ powder is dispersed in the RM solution, the amount of oxidizable RMs increases. In this case, the limiting current in the LSV profile reflects the regeneration of RMs, indicating the reaction rate between oxidized RMs and Li_2_O_2_. The results demonstrated that RMs with higher redox potentials generally exhibited better kinetics, implying the existence of a potential trade-off between energy efficiency and power capability in RMs-assisted batteries [91]. This trade-off suggested that not only thermodynamic aspects (i.e. the theoretical voltage) but also kinetic aspects (i.e. the chemical oxidation rate of Li_2_O_2_) must be earnestly considered while designing high-performance RMs.

## SUMMARY AND OUTLOOK

In this review, we summarize the operation mechanisms and properties of typical RMs for Li–O_2_ batteries, including organic, organometallic and inorganic compounds. Moreover, we discuss the main challenges associated with RMs-assisted Li–O_2_ batteries. Although several pioneering investigations have been performed to understand RMs-assisted Li–O_2_ batteries, notable advances are still desired to meet the requirements for practical applications. We also outline several possible research directions for advanced RMs and hope that our perspectives would contribute to the future development of RMs-assisted Li–O_2_ batteries. Concretely, the outlook will be propagated according to the following five aspects: understanding the oxidation kinetics of Li_2_O_2_ with RMs, regulating the molecular structure of RMs, optimizing the components of RMs-assisted Li–O_2_ batteries, analysing the catalytic efficiency of RMs and exploring the guideline for seeking new RMs.

- The most vexing obstacle is the kinetics of Li_2_O_2_ oxidation by RMs that need to be further studied. To date, there is relatively little research on the kinetic feature of RMs-assisted charging. Besides, it is also unclear whether there is a relationship between the kinetics of the chemical decomposition of Li_2_O_2_ by RMs and the kinetics of the electrochemical oxidation of RMs. Due to the complexity of Li–O_2_ batteries, involving gas, liquid and solid phases, traditional kinetic analytic methods are unsuitable. An appropriate electrochemical model is expected to overcome this obstacle and provide a guide for exploring the factors on reaction kinetics. Moreover, most current research focuses on understanding and optimizing the OER RMs. Only a few systematic studies were performed based on a general standard for an ideal ORR RM, which has severely hindered the development of the ORR RMs due to the lack of deep understanding.
- An ideal RM is supposed to be highly soluble, fully reversible and stable against active oxygen species. It should also yield proper redox potential and high diffusion coefficient. In addition, under the aim of practical applications, all the discussed RMs should have low cost and little toxicity. As discussed above, the physiochemical properties of RMs greatly depend on their molecular structure and operational environment. Rationally modifying the molecular structure of RMs may enable to address some awkward problems, such as the deterioration of RMs, shuttle effect and lower solubility. Furthermore, adjusting the RM diffusion kinetics may provide a new sight on the oxidation kinetics of Li_2_O_2_ by RMs.
- Reasonable match with the battery components is expected to achieve synergy and further improve battery performance. Engineering cathodes with abundant channels can provide efficient transport pathways for O_2_ and all redox-active species, which can realize a Li–O_2_ battery with larger capacity, better rate capability and longer cyclability. Besides, an electrolyte with low viscosity is beneficial to the diffusion of RMs. Notably, when RMs oxidize Li_2_O_2_, RMs may also oxidize or reduce the solvent. Side products from the decomposition of electrolytes and electrodes would block the O_2_-evolving interface. Therefore, improving the stability of electrodes and electrolytes should proceed in parallel with the efforts described herein. High concentration electrolytes (HCEs) have led to significant stability improvement in various electrochemical fields. The salts with high concentration in HCEs can coordinate with most solvent molecules and thus increase the stability of electrolytes without or with limited unstable free solvent molecules. As a result, the parasitic products associated with electrolytes are significantly reduced, thereby enhancing the transport current of the cathode and the accessibility of RMs to Li_2_O_2_ products, and ameliorating the catalytic efficiency of RMs. Besides, the HCEs can greatly improve the stability of Li-metal anodes because of the construction of an electrochemically stable SEI layer, which is expected to relieve the ‘redox shuttle’ of RMs.
- Although the RMs did facilitate the formation and decomposition of Li_2_O_2_, the overpotential observed from discharge or charge curves only provided partial information about the suitability of RMs in Li–O_2_ batteries. Some possible undesired reactions, widely observed as the detrimental decomposition of electrodes and electrolytes, might be missing in the unilateral electrochemical assessments. In addition, both the shuttle effect and stability issues for RMs confuse the precise assessment of the effectiveness of RMs. Any claim about the true catalytic effectiveness of RMs in Li–O_2_ batteries is inadequate without quantitative measurement. Therefore, multiple quantitative analyses are urgently required to investigate the yield of Li_2_O_2_, oxygen consumption and evolution. The appropriate measurement techniques could provide clear interpretation of the catalytic efficiency of RMs.
- Although numerous RMs have been investigated and applied, the general principles of seeking and designing a new type of RMs remain a mystery. Kang *et al.* suggested using ionization energy (IE) as a key indicator for designing RMs, where specific organic molecules with a certain range of IE values (5.8–6.8 eV) can be utilized as RMs in Li–O_2_ batteries [92]. Regretfully, this descriptor can only be applied to organic molecules, whereas their stability is slightly worse than that of inorganic RMs. It is challenging to explore excellent RMs, taking into account all aspects, including stability, redox potential, diffusion kinetics and catalytic activity, which may be troublesome to appraise owing to harsh experimental conditions. High-throughput computational screening can be performed on basis of ab initio calculations on candidate materials with a few physical parameters, to address all the above-mentioned problems at the same time. Furthermore, it is meaningful to identify a more general design principle to enable efficient searching for RMs, which would be beneficial for simplifying subsequent experimental procedures.

Objectively speaking, employing RMs is the most promising approach to tackle the sluggish reaction kinetics of Li–O_2_ batteries, although it is unlikely that all the problems in Li–O_2_ batteries can be addressed with RMs at the same time. More advanced experimental, computational and applied investigations are needed to advance the practical development of RMs-assisted Li–O_2_ batteries. The current status of practical applications of Li–O_2_ batteries seems extremely challenging. Major drawbacks, such as Li dendrite growth, electrolyte decomposition, unstable electrodes and operation in pure oxygen, prevent the progress. Future work towards practical Li–O_2_ batteries should primarily focus on the following three aspects. (i) Fundamental mechanisms underpinning Li–O_2_ electrochemistry. Performing theoretical modeling of the reactions between oxygen species and battery components, and combining electrochemical measurements with spectroscopic methods and online technology can identify possible electrochemical and chemical reactions in Li–O_2_ batteries. In addition, follow-up research should also provide some additional electrochemical performances, including self-discharge rate, performance at different temperatures and safety issues. (ii) Further optimization of battery components. It is generally accepted that the current electrodes and electrolytes, as well as cell structures, are far from real applications. Cathode materials with more stability, lower cost and higher catalytic activity play an important role in determining the Li–O_2_ battery performance. Besides, similarly to other Li-metal-based batteries, the safety issue of Li-metal anodes is also unavoidable. The research progress of Li-metal anodes in other batteries is helpful to the development of Li–O_2_ batteries. Especially, the influence of oxygen species (O_2_, O_2_^–^, LiO_2_ and ^1^O_2_) on Li-metal anodes must be considered in future research. Meanwhile, electrolyte evaporation also needs to be addressed by optimizing the battery structure or employing polymer and solid electrolytes [93]. Though RMs play a catalytic role by dissolving in electrolytes, their applications to Li–O_2_ batteries with polymer and solid electrolytes are still needed but extremely difficult. Anchoring RMs at the electrode surface or introducing RMs to the working gas outside the assembled battery might be considered in the future, which can overcome the limitation of dissolution characteristics while maintaining the catalytic function of RMs. (iii) True Li–air batteries. Most reported Li–O_2_ batteries are operated under a pure-oxygen environment. However, to achieve a true ‘Li–air’ battery in the future, the battery should eventually be operated in ambient air. Although an appropriate amount of impurity gas can improve the battery performance, the fickle external environment have made it difficult to achieve Li–air batteries until now. Designing an O_2_ selective membrane is a feasible strategy to ensure that the battery works under a constant O_2_ atmosphere, thereby indirectly realizing the operation of Li–O_2_ batteries under ambient conditions. Furthermore, more research should be devoted to understanding the influence of other gases in air on battery performances and then developing high-efficiency multifunctional catalysts to simultaneously catalyse the reversible reactions of other gases, especially CO_2_ and water, eventually realizing true Li–air batteries.
